# Unsupervised Coverage Sampling to Enhance Clinical Chart Review Coverage for Computable Phenotype Development: Simulation and Empirical Study

**DOI:** 10.2196/72068

**Published:** 2025-11-27

**Authors:** Zigui Wang, Jillian H Hurst, Chuan Hong, Benjamin Alan Goldstein

**Affiliations:** 1 Department of Biostatistics and Bioinformatics, Duke University School of Medicine, Duke University, 2424 Erwin Road, 9023 Hock Plaza, Durham, NC, 27705, United States, +1 919-691-5011; 2 Department of Pediatrics, Duke University School of Medicine, Duke University, Durham, NC, United States

**Keywords:** electronic health records, EHR, chart review sampling, coverage metric, computable phenotypes

## Abstract

**Background:**

Developing computable phenotypes (CP) based on electronic health records (EHR) data requires “gold-standard” labels for the outcome of interest. To generate these labels, clinicians typically chart-review a subset of patient charts. Charts to be reviewed are most often randomly sampled from the larger set of patients of interest. However, random sampling may fail to capture the diversity of the patient population, particularly if smaller subpopulations exist among those with the condition of interest. This can lead to poorly performing and biased CPs.

**Objective:**

This study aimed to propose an unsupervised sampling approach designed to better capture a diverse patient cohort and improve the information coverage of chart review samples.

**Methods:**

Our coverage sampling method starts by clustering by the patient population of interest. We then perform a stratified sampling from each cluster to ensure even representation for the chart review sample. We introduce a novel metric, nearest neighbor distance, to evaluate the coverage of the generated sample. To evaluate our method, we first conducted a simulation study to model and compare the performance of random versus our proposed coverage sampling. We varied the size and number of subpopulations within the larger cohort. Finally, we apply our approach to a real-world data set to develop a CP for hospitalization due to COVID-19. We evaluate the different sampling strategies based on the information coverage as well as the performance of the learned CP, using the area under the receiver operator characteristic curve.

**Results:**

Our simulation studies show that the unsupervised coverage sampling approach provides broader coverage of patient populations compared to random sampling. When there are no underlying subpopulations, both random and coverage perform equally well for CP development. When there are subgroups, coverage sampling achieves area under the receiver operating characteristic curve gains of approximately 0.03‐0.05 over random sampling. In the real-world application, the approach also outperformed random sampling, generating both a more representative sample and an area under the receiver operating characteristic curve improvement of 0.02 (95% CI −0.08 to 0.04).

**Conclusions:**

The proposed coverage sampling method is an easy-to-implement approach that produces a chart review sample that is more representative of the source population. This allows one to learn a CP that has better performance both for subpopulations and the overall cohort. Studies that aim to develop CPs should consider alternative strategies other than randomly sampling patient charts.

## Introduction

Electronic health records (EHR) data are widely used in clinical research. While they contain dense, often granular information on a patient’s health status, they also pose challenges for clinical studies since they lack explicit documentation for the reason for the health care encounter (eg, admission due to infection). In principle, the problem list, which provides a historical listing of previous health problems, can be used to identify chronic conditions, though it is often unreliable [1,2]. Similarly, fields such as discharge diagnosis may not accurately represent the reason a patient had a visit. Instead, information from diagnosis codes, laboratory test results, and prescriptions or administered medications is used to indicate the presence of a specific clinical condition [3-5]. This is a well-known challenge in working with EHR data and has led to the growth of computable phenotypes (CPs). CPs are algorithms, typically Boolean, though sometimes probabilistic, that use multiple sources of clinical data—such as diagnoses, laboratory results, and medication records—to infer the clinical condition of a patient or the reason for a visit [6-8].

**Figure 1.**
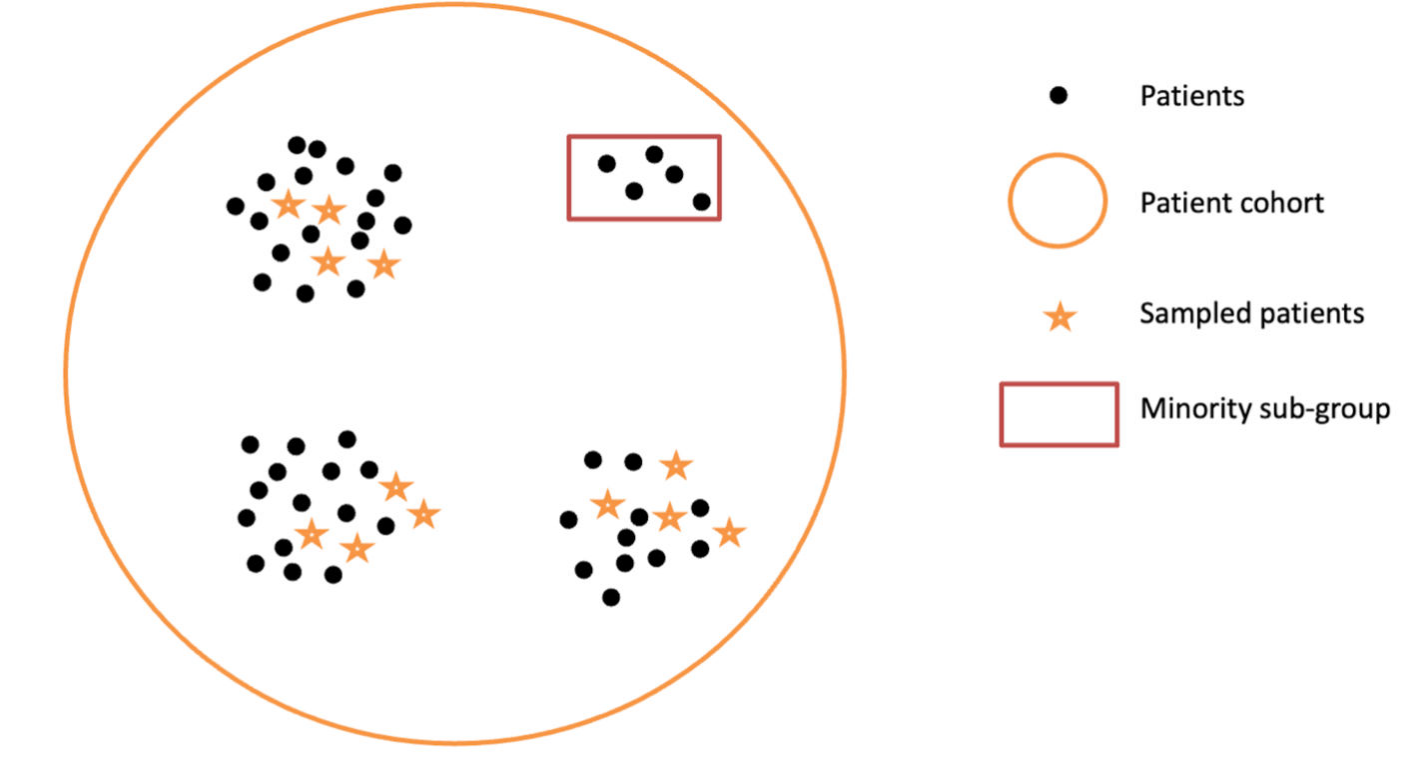
Example of the impact of random sampling on the representation of patient subgroups. The black dots represent unsampled patients; stars represent the sampled patients. The red box represents a subgroup that was missed by random sampling.

Creating CPs is a multiphase process that often requires significant collaborative effort from clinicians and informaticians [5,9]. One of the key components in CP development is the creation of a set of “gold standard” outcome labels. The outcome labels are typically generated based on manual review of a subset of eligible patient charts, which can require significant time [6-8,10]. The set of charts that are used to develop these gold-standard labels is usually sampled randomly [11]. While random sampling will, on average, produce a representative view of the population of interest, since one usually wants to review only a small number of charts, random sampling may not adequately represent the complete range of disease presentations or patient demographics. In the scenario shown in Figure 1, subgroups that have a rarer presentation within the larger data set (eg, rarer presentations of the disease of interest and disease presentation in minority subgroups) are less likely to be adequately covered based on random sampling. In this case, much larger sample sets are necessary to find a meaningful number of charts from people from these subgroups [12]. In such scenarios, random sampling strategies might not be effective in generating a sample covering all subgroups for chart review purposes and result in a CP that does not accurately capture the heterogeneity of the condition of interest. This can lead to a CP that performs worse for those subpopulations.

In recent years, various methods have been developed to enhance the coverage of labeled data [13-16]. However, these methods either rely on external population resources or focus solely on maximizing demographic coverage, which do not fully align with the chart review objective. Among these methods, active learning is one such approach that iteratively selects the most informative samples for labeling, aiming to optimize model performance with a minimal amount of data [17,18]. However, active learning typically requires an initial set of labeled data to train the model and guide the selection process [19]. Moreover, active learning methods are typically focused on identifying the samples that will provide the most leverage on the final model, as opposed to the ones that would best capture the diversity of the patient cohort [18-21].

In this paper, we propose a process for selecting medical charts for review to generate gold standard labeling when constructing CPs. The goal of this method is to ensure that our selection captures the diversity of the full patient cohort. To achieve this, we propose a clustering-based process to generate potential samples. We then introduce a novel metric to identify the most representative sample that should be used for label generation. By enhancing the information coverage of the training sample, our approach is expected to yield a better performing CP for both subgroups and the full patient cohort. To illustrate this approach, we use simulation methods coupled with a real-world data example to demonstrate how this novel sampling approach can match or even surpass the performance of random sampling.

## Methods

### Sampling Approach 

**Figure 2.**
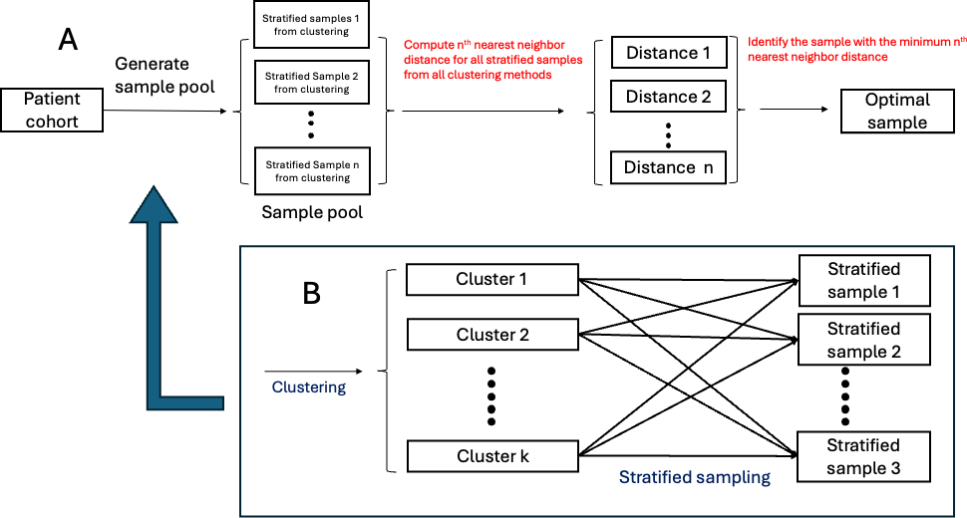
Diagram illustrating our sampling approach. (A) procedures for general coverage sampling; (B) procedures for generating the sample pool.

The overall methodological approach is illustrated in Figure 2. We start by considering a study cohort for whom we want to create labels for the presence or absence of a condition of interest (eg, diabetes, cause-specific admission). We presume that our study cohort is large enough that we do not want to review and label all patient charts. Instead, we want to generate an optimal chart review sample*,* from which we will develop or “learn” a CP. In this paper, our analytic task is to determine how to best identify that sample. We propose that the best sample is one that maximizes coverage of the cohort, providing information about all of the subgroups that compose the cohort (Figure 2A). In other words, the sample should be equally representative of each subgroup, rather than merely reflecting the source population distribution. To assess coverage, we define a novel metric, described below. A variety of methods can be used to generate the sample pool. In this study, we propose using a stratified sampling framework, in other words, clustering the data and then sampling from these clusters (Figure 2B). By identifying and then sampling from clusters, we hypothesize that we will be able to represent different patient subgroups, making the chart review sample more reflective of the entire patient cohort.

#### Optimal Sample Generation

After defining a cohort of interest, we start by clustering the individual patient records. As illustrated in our real data example, our groupings are driven by clinical factors, so we only use clinical features (ie, not demographic factors) to conduct the clustering. We then sample records randomly from each of the clusters. For example, if we prespecify that we want to review 100 charts, and we generate 4 clusters, we would sample 25 records from each cluster. While a variety of clustering algorithms can be used, we suggest hierarchical clustering. The nested cluster structure provided by hierarchical clustering is reflective of our proposed interpretation that the cohort consists of patient subgroups. For comparison, we also present results from K-means clustering. Notably, with a sufficiently large number of replications, we expect different clustering methods to generate similar optimal samples, resulting in comparable representative samples.

#### Coverage Assessment

A primary step is assessing data coverage. To do so, we propose a novel metric that measures the coverage of the sample for the full data cohort. We define the n^th^ nearest neighbor distance as:


$$n^{th}\;nearest\;neighbor\;distance=\;\sum_{i=1}^{N}d_{i}^{n}$$

For each person, *i*, in the study cohort of size N, we calculate the Euclidean distance, *d*, to each person from the sampled set. $d_{i}^{n}$ is the distance between the $i^{th}$ person in the cohort, to the $n^{th}$ nearest sampled person. For example, the 5th nearest neighbor distance refers to the sum of the distance between every patient *i* and its 5th closest sampled person. After generating the sample pool, we calculate the distance for each individual in the patient cohort to the $n^{th}$ nearest sampled person. We choose the chart review sample with the lowest $n^{th}$ nearest neighbor distance. The intuition for the $n^{th}$ nearest neighbor distance is to ensure that for each person in the full cohort, there is someone in the chart review sample that is “near” or representative of them. This should result in greater coverage for underrepresented subgroups and phenotypes compared to random sampling. For example, for a disease that can present clinically in a variety of ways (eg, diabetes), if a rarer presentation is not represented in the chart sample, then for a person i from this minority group, the closest sampled individuals will be in other subgroups, resulting in a larger $n^{th}$ nearest neighbor distance. Moreover, sampling to minimize the $n^{th}$ nearest neighbor distance will not adversely impact the majority presentation since group members will still have representative samples.

Our coverage sampling process can be summarized as follows:

1. Cluster the dataset based on clinical factors across a range of k clusters.
2. Conduct stratified sampling with a specified sample size across the clusters multiple times to generate the sample pool.
3. Calculate the $n^{th}$ nearest neighbor distance for each sample set in the sample pool and identify the sample with the minimal $n^{th}$ nearest neighbor distance.

The primary tuning parameter is n. This can be prespecified by the user, or *n* can be assessed over a range of values, and taking the mean distance. As we show below, the approach is not very sensitive to the choice of n. Although the results presented are based on Euclidean distance, given the mixed data types of the EHR data, we also assessed Manhattan and Gower distances and found that they had minimal impact on the final results.

#### Assumptions

The primary assumption of this procedure is that we have a broad cohort from which to sample that fully captures all individuals with the condition from which we wish to define a CP. Meaning, our identified patient cohort (ie, our denominator) has perfect sensitivity for the outcome of interest, and the analytic challenge is improving the specificity of the CP.

#### Evaluation Criteria

**Figure 3.**
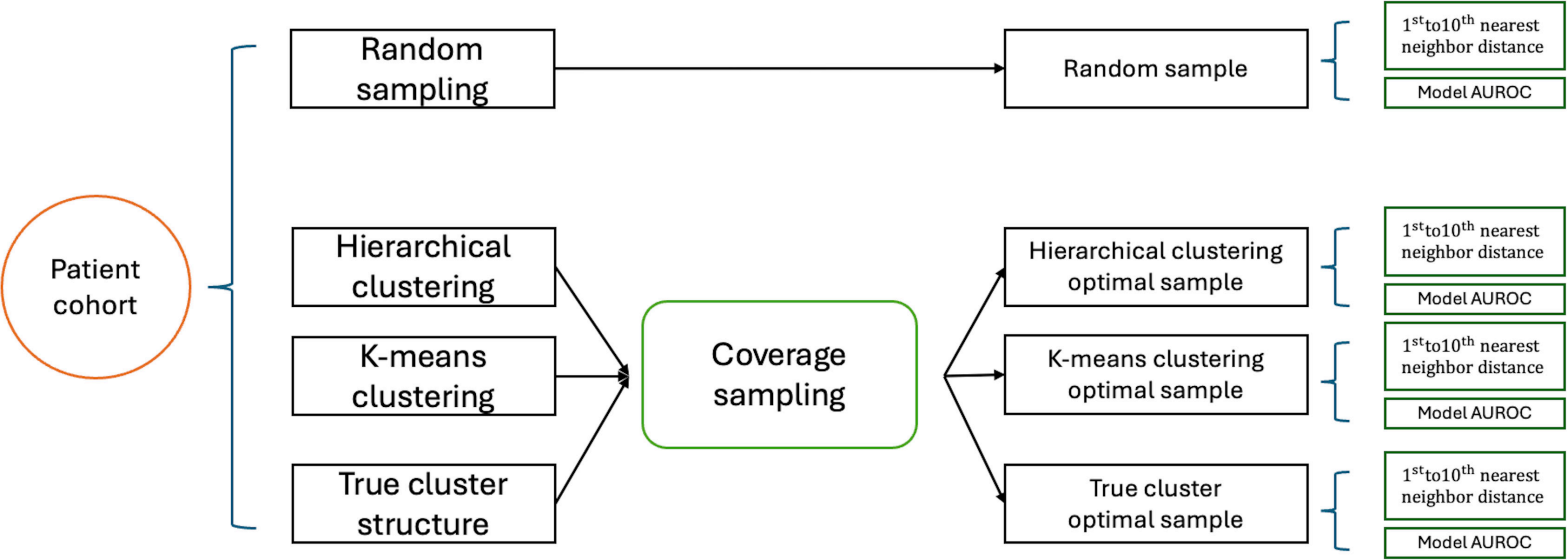
Diagram to show the evaluation criteria of sample quality. The coverage sampling refers to the sampling procedure outlined in Figure 2. AUROC: area under the receiver operating characteristic curve.

As shown in Figure 3, we assess the quality of a selected sample for chart review in 2 ways: cohort coverage and CP performance. For cohort coverage, we compare the $n^{th}$ nearest neighbor distance, with samples exhibiting smaller distances considered more representative of the study cohort. For CP performance, we train a classification model using a sample derived from either our proposed coverage sampling or random sampling methods. All the unsampled patients are regarded as the test dataset. We evaluate the efficacy of these models by comparing the area under the receiver operating characteristic curve (AUROC) using the test dataset. Samples that yield models with higher AUROC values are considered to be better.

### Simulation Study

We conduct a simulation study to evaluate the efficacy of coverage sampling outlined above. We sample 120 patients from 250 simulated datasets with a size of 10,000 and 10 characteristic variables by both random sampling and coverage sampling. Across the datasets, we generate 4 clusters and create a sample with different proportions of each cluster: (simulation set 0: 1.0,0,0,0; simulation set 1: 0.25, 0.25, 0.25, 0.25; simulation set 2: 0.1, 0.3, 0.3, 0.3; simulation set 3: 0.1, 0.1, 0.4, 0.4 and simulation set 4: 0.1, 0.1, 0.1, 0.7). These samples can be interpreted as: no underlying cluster structure, equally distributed subgroups, 1 minority subgroup, 2 minority subgroups, and one majority group. The initial 2 simulation sets (sets 0‐1) serve as baselines, where either no underlying cluster exists or all clusters are of equal size. The subsequent simulations (sets 2‐4) delve into more complex scenarios, incorporating minority subgroups to assess their impact on the representation of subgroups within the samples.

To generate the clustered data, we used the R package fungible [13], which uses the following model:


$$X=D_{j}B+e$$

Where X is the matrix of simulated observations, where each row representing an observation and each column representing a variable; $D_{j}$ is a matrix of indicator for cluster j, identifying the membership of observation within this cluster; B is a matrix that represents the correlation between the cluster membership and observation scores), and e represents the deviations that generated from a mixture distribution.

To generate outcomes (ie, phenotypes to be derived), we apply the following model:


$$logit\left(P\left(event=1\right)\right)=\alpha_{0}+\sum_{j=1}^{k}I\left(cluster_{i}=j\right)\alpha_{j}+\sum_{l=1}^{p}X_{il}\beta_{l}$$

Where $P\left(event=1\right)$ represents the probability of $i^{th}$ encounter outcome equal to 1. $I({cluster}_{i}=j)$ is the indicator of whether the $i^{th}$ encounter belongs to $j^{th}$ cluster or not. *X* is the design matrix where each row represents one encounter, and each column represents one explanatory variable. $\alpha_{0}$, $\alpha_{i}$, and $\beta_{l}$ are the intercept and main effects corresponding to $I({cluster}_{i}=j)$ and *X*. To more accurately reflect real-world conditions, only half of the explanatory variables were incorporated into the generation of the outcome variables, treating the remaining variables as noise (with respect to the outcome).

For each simulated dataset, we assessed information coverage and model performance across 3 samples. First, using the procedure described in the optimal sample generation section, we averaged the 1st to 10th nearest neighbor distances to obtain a hierarchical-cluster-based sample of size 120 and a k-means-cluster-based sample of size 120. For comparison, we selected 120 random samples and 120 that were sampled from the true underlying clusters. In this manuscript, we refer to the 4 samples as hierarchical, k-means, random, and truth. All data not included in these samples were retained as test data for further analysis. For the model performance comparison, we used each of the 4 derived samples (hierarchical cluster coverage, k-means cluster coverage, random, truth) to fit a logistic regression model to learn a probabilistic CP. For the scenario without an underlying cluster structure, only hierarchical cluster coverage, k-means coverage, and random sampling results are presented, as there is no true cluster structure to compare against. We computed the AUROC to evaluate the model’s performance and averaged the performance over 50 iterations.

### Real-World Data Application

Our application is motivated by our previous work to develop a CP for a hospital admission due to COVID-19. During the height of the COVID-19 pandemic, hospitals tested all patients for SARS-CoV-2. Work by us [14] and others [15] has indicated that up to 38% of patients who tested positive for SARS-CoV-2 upon admission were admitted for reasons other than COVID-19. Therefore, a CP for admission due to COVID-19 would need to be more complex than simply a positive SARS-CoV-2 test. Our goal then is to define a sample of patients for chart review, in aid of learning a CP for admission due to COVID-19. Since COVID-19 patients could have different presentations, we hypothesize that our coverage sampling approach would be better for learning a CP.

#### Data Source

We abstracted data from the Duke University Health System EHR system. Duke University Health System consists of 3 hospitals on a common, EPIC-based EHR system. The clinical data are organized into a research-ready datamart, based on the PCORnet Common Data Model [16].

#### Source Cohort

Our study cohort consisted of all patients with an inpatient admission and a positive test for SARS-CoV-2 from March 2020 to March 2023 (when routine testing stopped). This definition has perfect sensitivity, but poor specificity, for capturing admissions due to COVID-19. Following our previous work, we split this cohort into training and testing data. The testing data consisted of 441 patients admitted from January 16 to 22, 2022 and were already chart reviewed for operational purposes. Additional information regarding the testing data can be found in [14]. The training data consisted of the other 7743 unlabeled patients with positive SARS-CoV-2 tests from 2020‐2023.

#### Features Used

For coverage sampling and CP generation, we used 46 clinically relevant features such as encounter characteristics (encounter type, admitting source, and discharge disposition), diagnoses, laboratory tests conducted, and medications administered. Table S1 in Multimedia Appendix 1 provides full details on features used. While we extracted demographic characteristics, we did not include these in the sampling or CP development steps.

#### Sampling and Outcome Labeling

We generated 2 samples of 100 using coverage and random sampling from the training dataset of 7743 patients. For the coverage sampling, we used hierarchical clustering and identified the cluster structure that minimized the *1st* nearest neighbor distance. An infectious disease specialist (JHH) chart reviewed and labeled the encounter as due to COVID-19 or related sequela, or not.

#### Method Evaluation

We compare the patient characteristics for the samples that were selected from each sampling approach. Then, using the criteria defined above, we evaluate the coverage of the sample of the full cohort. Finally, we used each sample to learn a probabilistic CP based on a least absolute shrinkage and selection operator logistic regression. We evaluated each version on the independent test data.

All analyses were conducted in R version 4.3.2. The source code used in these experiments is available at GitHub [22].

### Ethical Considerations

This study was approved and declared exempt by the Duke School of Medicine IRB, protocol Pro00109397 (9/14/2021). We used a limited analytical dataset within a secure computing environment, and patients did not receive any compensation.

## Results

### Evaluation of Sampling Methods Using Simulated Data

**Figure 4.**
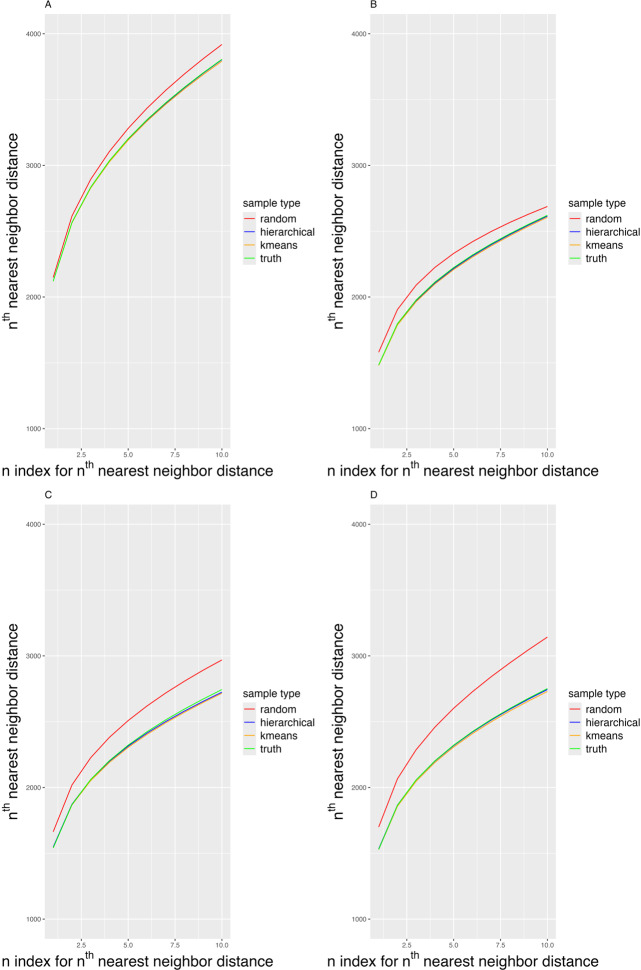
From 1st to 10th nearest neighbor distance mean across 200 simulated data samples for 4 cluster ratios. The red line represents the random sample; the blue line represents the coverage sampling based on hierarchical cluster; the orange line represents the coverage sampling based on k-means clustering; the green line represents the coverage sampling based on the true cluster. (A) All simulated data follows the baseline cluster ratio (0.25,0.25,0.25,0.25). (B) All simulated data follows cluster ratio (0.1,0.3,0.3,0.3). (C) All simulated data follows cluster ratio (0.2,0.2,0.4,0.4). (D) All simulated data follows cluster ratio (0.1,0.1,0.1,0.7).

Figure 4 presents the mean 1st to 10th nearest neighbor distances for both random and coverage sampling methods across 4 distinct scenarios. Figure 4A illustrates a baseline scenario where all clusters are of equal size, while Figure 4B-D depict scenarios with 1, 2, and 3 minority subgroups, respectively. In each scenario, the coverage samples consistently exhibit smaller nearest neighbor distances compared to those from random samples. As minority subgroups are incorporated into the simulated cohort, the advantage of the coverage sample over a random sample increases. Notably, the distances in the hierarchical and k-means-clustered coverage samples are closely aligned with those observed in true cluster configurations, indicating similar coverage of the cohort.

After generating the samples, we used the data to learn a probabilistic CP and tested its performance. Table 1 presents the mean 1st to 10th nearest neighbor distance and the mean logistic model’s AUROC between a random sample and coverage samples generated using hierarchical clustering, k-means clustering, and a true cluster structure of size 120 across 250 simulated datasets. Values highlighted in asterisk (Table 1) indicate AUROCs that are significantly higher than those of the random sample at the 0.05 significance level. The visualization of the AUROC results is shown in Figure S1 in Multimedia Appendix 1. Consistent with coverage results, in the baseline scenario without any minority subgroup or any underlying cluster structure, coverage samples exhibit similar AUROC compared to random samples. However, with the introduction of a minority subgroup, the coverage samples based on hierarchical, k-means, and true cluster structures all produced significantly higher AUROC values compared to random samples. Additionally, we observed that the coverage samples using hierarchical clustering and k-means clustering exhibited similar performance, suggesting that the choice of clustering method has minimal impacts on coverage sampling, provided there are sufficient repetitions.

**Table 1.** Comparison of mean distance and area under the curve between random sample, sample based on coverage samples based on hierarchical clustering, k-means clustering, and true cluster.

Cluster ratio and sample type	Mean of 1st-10th nearest neighbor Distance	Overall AUROC^a^ (95% CI)	Subgroup 1 AUROC (95% CI)	Subgroup 2 AUROC (95% CI)	Subgroup 3 AUROC (95% CI)	Subgroup 4 AUROC (95% CI)
(1.0,0,0,0)						
Random	3877.728	0.806 (0.803-0.809)	N/A^b^	N/A	N/A	N/A
Hierarchical	3802.404	0.805 (0.801-0.808)	N/A	N/A	N/A	N/A
K-means	3823.452	0.805 (0.802-0.809)	N/A	N/A	N/A	N/A
(0.25,0.25,0.25,0.25)						
Random	3257.498	0.751 (0.747-0.755)	0.735 (0.732-0.738)	0.739 (0.735-0.743)	0.731 (0.728-0.735)	0.733 (0.730-0.736)
Hierarchical	3170.708	0.751 (0.747-0.756)	0.734 (0.731-0.738)	0.739 (0.735-0.742)	0.731 (0.727-0.734)	0.732 (0.729-0.735)
K-means	3163.75	0.752 (0.748-0.757)	0.735 (0.732-0.738)	0.738 (0.734-0.742)	0.731 (0.727-0.734)	0.732 (0.729-0.735)
Truth	3173.694	0.748 (0.744-0.753)	0.731 (0.727-0.735)	0.735 (0.731-0.738)	0.727 (0.724-0.731)	0.729 (0.725-0.732)
(0.1,0.3,0.3,0.3)						
Random	2293.954	0.691 (0.678-0.703)	0.706 (0.694-0.717)	0.607 (0.596-0.618)	0.609 (0.599, 0.618)	0.601 (0.593-0.610)
Hierarchical	2192.928	0.742^c^ (0.731-0.753)	0.750^c^ (0.741-0.760)	0.633^c^ (0.623-0.643)	0.638^c^ (0.630-0.647)	0.629^c^ (0.620-0.638)
K-means	2183.826	0.740^c^ (0.729-0.752)	0.746^c^ (0.737-0.755)	0.632^c^ (0.623-0.642)	0.636^c^ (0.628-0.644)	0.628^c^ (0.620-0.636)
Truth	2197.208	0.739^c^ (0.727-0.751)	0.747^c^ (0.736-0.757)	0.631^c^ (0.621-0.642)	0.634 (0.625-0.643^c^)	0.622^c^ (0.613-0.632)
(0.1,0.1,0.4,0.4)						
Random	2478.755	0.747 (0.737-0.757)	0.746 (0.737-0.756)	0.743 (0.734-0.753)	0.619 (0.612-0.626)	0.617 (0.609-0.626)
Hierarchical	2286.317	0.778^c^ (0.769-0.788)	0.774^c^ (0.767-0.781)	0.769^c^ (0.762-0.776)	0.636^c^ (0.630-0.641)	0.635^c^ (0.628-0.642)
K-means	2276.429	0.775^c^ (0.764-0.786)	0.774^c^ (0.767-0.781)	0.771^c^ (0.764-0.778)	0.633^c^ (0.628-0.639)	0.634^c^ (0.627-0.640)
Truth	2292.119	0.782^c^ (0.773-0.791)	0.778^c^ (0.772-0.784)	0.773^c^ (0.767-0.779)	0.639^c^ (0.633-0.644)	0.637^c^ (0.630-0.644)
(0.1,0.1,0.1,0.7)						
Random	2584.656	0.731 (0.718-0.745)	0.740 (0.730-0.750)	0.743 (0.735-0.752)	0.739 (0.730-0.747)	0.586 (0.580-0.592)
Hierarchical	2287.864	0.769^c^ (0.756-0.782)	0.776^c^ (0.771-0.782)	0.776^c^ (0.770-0.781)	0.772^c^ (0.767-0.777)	0.601^c^ (0.595-0.607)
K-means	2276.252	0.775^c^ (0.762-0.788)	0.777^c^ (0.771-0.783)	0.774^c^ (0.769-0.780)	0.771^c^ (0.765-0.776)	0.600^c^ (0.594-0.606)
Truth	2290.974	0.772^c^ (0.759-0.785)	0.780^c^ (0.774-0.786)	0.778^c^ (0.773-0.783)	0.774^c^ (0.769-0.780)	0.601^c^ (0.596-0.607)

a AUROC: area under the receiver operating characteristic curve.

b N/A: not applicable.

c indicates that the AUROC is significantly higher than that of random sampling method at the .05 significance level.

### Evaluation of Sampling Methods Using Real-World Data

**Figure 5.**
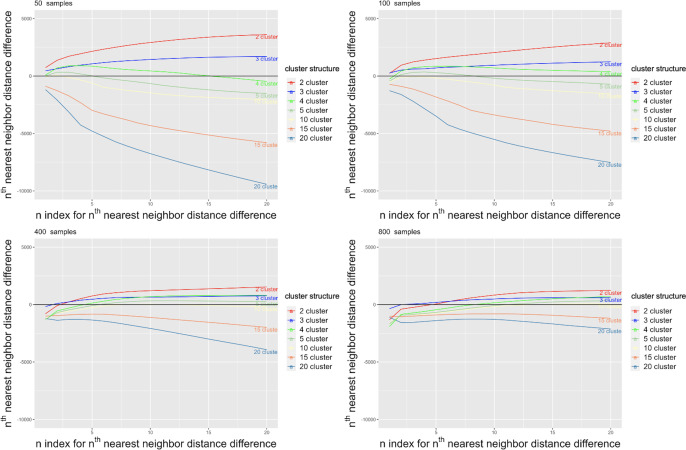
Mean n^th^ nearest neighbor distance difference (random sample distance–coverage sample distance) over 100 replications for real-world data.

In real-world data, the true number of clusters or patient subgroups is unknown. We therefore explored a range of potential cluster structures, including 2, 3, 4, 5, 10, 15, and 20 cluster structures. Based on simulated data results, with enough replications, the choice of clustering method does not impact our sampling approach; thus, we only evaluated hierarchical clustering in the real-world data analysis. Figure 5 shows the difference in the mean 1st to 20th nearest neighbor distances in samples generated using the coverage and random samples (ie, coverage sample $n^{th}$ nearest neighbor distance subtracted from random sample $n^{th}$nearest neighbor distance). The results demonstrate that for smaller sample sizes (50 and 100), samples drawn from structures with 2, 3, and 4 clusters provide a more accurate representation than their random counterparts. However, as the chart review sample size increases from 400 to 800, the $n^{th}$ nearest neighbor distance of the coverage samples aligns more closely with that of random samples. It is noteworthy that samples derived from 10, 15, and 20 cluster structures perform less effectively across all sample sizes.

We conducted a chart review of the 100 random samples and 100 coverage samples. Our coverage sampling approach selected a sample based on 1st nearest neighbor distance. Assessing the clusters using nearest neighbor distance indicated that 2 clusters were the optimal cluster structure. Major clinical differences between the 2 clusters included the C-Reactive protein test, D-dimer test, and BMI (Table S1 in Multimedia Appendix 1). Moreover, while clustering was performed using only clinical variables, the resulting clusters also exhibited meaningful demographic differences, with cluster 1 consisting of an older population compared to cluster 2 (50% vs 25% individuals older than 65 y old). Additional details regarding the cluster characteristics are provided in Table S1 in Multimedia Appendix 1. Table 2 presents the demographic characteristics of the full 7743 patient cohort, as well as the demographics of the coverage and random samples. The standardized mean differences of the random sample and coverage sample are also shown. Notably, the coverage sample includes a higher percentage of young adults (24/100) compared to the random sample (11/100), with other demographic variables showing similar prevalence patterns in both samples.

**Table 2.** Demographic characteristics of real-world data, full sample, coverage sample, and random sample.

Characteristics	Full sample	Test dataset	Coverage sample	Random sample	Random versus cluster SMD^a^
Sample size	7743	441	100	100	
Male sex, n (%)	3737 (48.3)	221 (50.1)	43 (43.0)	47 (47.0)	0.080
Age (years), n (%)					0.391
Children (0‐18)	307 (4.0)	11 (2.5)	3 (3.0)	2 (2.0)	
Young adult (18-35)	995 (12.9)	63 (14.3)	24 (24.0)	11 (11.0)	
Middle adult (35-65)	3015 (38.9)	177 (40.1)	38 (38.0)	38 (38.0)	
Older adult (>65)	3426 (44.2)	190 (43.1)	35 (35.0)	49 (49.0)	
Race and Ethnicity, n (%)					0.131
Hispanic	833 (10.8)	30 (6.8)	13 (13.0)	15 (15.0)	
Non-Hispanic Black	2999 (38.7)	211 (47.8)	44 (44.0)	39 (39.0)	
Non-Hispanic white	3572 (46.1)	187 (42.4)	36 (36.0)	40 (40.0)	
Non-Hispanic Asian	110 (1.4)	1 (0.2)	3 (3.0)	2 (2.0)	
Other races	229 (3.0)	12 (2.7)	4 (4.0)	4 (4.0)	
Group primary payment					0.239
Private	3564 (46.0)	216 (49.0)	48 (48.0)	50 (50.0)	
Public	3215 (41.5)	176 (39.9)	42 (42.0)	35 (34.0)	
Self-pay	307 (4.0)	22 (5.0)	4 (4.0)	3 (3.0)	
Others	657 (8.5)	27 (6.1)	6 (6.0)	12 (12.0)	

a SMD: standardized mean differences.

After generating labels, we fit a least absolute shrinkage and selection operator logistic regression model to learn and test a CP. Table 3 presents the *1st* nearest neighbor distance for both the coverage sample and random sample, as well as the AUROC for the learned CP. Additionally, we plot the 1st to 20th nearest neighbor distances of coverage and random sample in Figure S2 in Multimedia Appendix 1.

Given that the true cluster structure is unknown, we report the AUROC at the demographic variable level. The 1st nearest neighbor distance and the AUROC results indicate that the coverage sample slightly outperforms the random sample. This pattern is also observed at the demographic feature level; however, these differences did not reach statistical significance. Nonetheless, in terms of magnitude, coverage samples demonstrate a notable improvement in the coverage of young adults compared to random samples. Of note, we found that increased coverage of demographic groups is not directly correlated with model performance. For example, the coverage and random samples both have similar proportions of males and females. However, the coverage sampling performs nominally better within each sex group. This supports not including demographics in the clustering step and relying on clinical drivers of differentiation. We further evaluated the performance of coverage sampling using additional model architectures, including random forest and XGBoost, for real-world data. Across all models, the coverage sampling approach consistently performed as well as, or better than, random sampling. More details can be found in Table S2 in Multimedia Appendix 1.

For very small subgroups, the performance of the coverage sample may vary. For instance, in the “Other race” group (n=12), the coverage sample shows a substantial AUROC improvement over random sampling (0.611 vs 0.925). In contrast, for the “Other payment” group (n=27), the coverage sample performs slightly worse than random sampling (0.805 vs 0.820).

**Table 3.** Mean area under the curve comparison between the coverage sample and the random sample on real-world data.

Characteristics (n)	Random sample AUROC^a^ (95% CI)	Cluster sample AUROC (95% CI)
First nearest neighbor distance	57777.82	54213.58
Overall	0.726 (0.680-0.772)	0.747 (0.701-0.793)
Sex		
Female (n=220)	0.724 (0.659-0.784)	0.763 (0.695-0.824)
Male (n=221)	0.725 (0.656-0.789)	0.730 (0.663-0.797)
Age (years), n (%)		
Children (0‐18; n=2)	0.609 (0.312-0.875)	0.656 (0.312-0.937)
Young adult (18 – 35; n=65)	0.789 (0.685-0.886)	0.867 (0.774-0.947)
Middle adult (35 – 65; n=179)	0.723 (0.652-0.793)	0.727 (0.647-0.797)
Older adult (>65; n=185)	0.669 (0.587-0.749)	0.673 (0.588-0.754)
Race and ethnicity, n (%)		
Hispanic (n=30)	0.828 (0.674-0.963)	0.850 (0.692-0.973)
Non-Hispanic Black (n=211)	0.730 (0.660,0.791)	0.736 (0.664-0.802)
Non-Hispanic white (n=187)	0.703 (0.629,0.778)	0.725 (0.658-0.798)
Non-Hispanic Asian (n=1)	N/A^b^	N/A
Other races (n=12)	0.611 (0.222-0.944)	0.925 (0.778-1)
Group primary payment		
Private (n=216)	0.698 (0.626-0.764)	0.737 (0.667-0.805)
Public (n=176)	0.743 (0.672-0.811)	0.736 (0.661-0.809)
Self-pay (n=22)	0.642 (0.423-0.857)	0.733 (0.485-0.923)
Others (n=27)	0.820 (0.641-0.961)	0.805 (0.623-0.950)

a AUROC: area under the receiver operating characteristic curve.

b N/A: Not applicable.

## Discussion

### Principal Findings

CPs are a key component of secondary research with EHR data [23,24]. A required step in CP development is conducting a manual chart review to establish a set of “gold-standard” labels to identify patients with and without the condition or outcome of interest. This manual review can be highly time-consuming [25,26]. Little work has been conducted on how to optimally select charts for review, with investigators most often using random chart selection [11]. This can lead to inefficiencies as potentially informative or edge cases can be missed. To address this concern, we have proposed a sampling strategy to select charts for review that captures the diversity of a population of interest. The key aspect of our method is identifying the optimal sample using a new metric that we have termed the $n^{th}$ nearest neighbor distance. We assessed our method using both simulated and real-world data, evaluating both the information coverage and CP performance. Our findings indicate that coverage sampling performs as well as, if not better than, random sampling. We recommend using a representative sample when developing a computable phenotype. Alternatively, if the phenotype has already been developed, it should be recalibrated using a representative sample of the population before deployment.

One of the motivations for this approach is the presumption that within any group of patients with a particular condition, there are patient subgroups that may have a different presentation of that condition. For example, while many patients with diabetes will have glycosylated hemoglobin test values >6.5% there will be some individuals with controlled diabetes and normal glycosylated hemoglobin values; however, these patients still have diabetes [27]. Such scenarios require the creation of complex CPs that can identify patients with diabetes who have a variety of disease presentations [28]. If these patient subgroups are small enough, a random selection of charts may not provide sufficient coverage of these subgroups to ensure that the CP performs equitably for all patient subgroups. As our results demonstrate, coverage sampling has its greatest impact in CP performance when minority subgroups are present. However, the presence of such minority subgroups is not a requirement for the method to perform well. In scenarios without minority subgroups, our method performs comparably to random sampling, highlighting the robustness of the approach. Moreover, even when there is no underlying cluster structure at all, coverage sampling performs as well as random sampling.

A novel aspect of our approach is the development of a metric, the $n^{th}$ nearest neighbor distance, to measure the coverage of a given sample. While previous methods have used nearest neighbor distances to detect and quantify spatial randomness, they have primarily focused on analyzing spatial patterns in populations [29]. Existing representativeness metrics, such as Simpson’s Diversity Index and Shannon’s Entropy, quantify overall variability across multiple demographic features [30]. Simpson’s Diversity Index measures the probability that 2 individuals randomly selected from a sample will belong to different categories, thereby emphasizing the dominance or evenness of group representation [31]. Shannon’s Entropy quantifies diversity by accounting for both the abundance and the evenness of the categories present, using information theory to assess the uncertainty in predicting the category of a randomly chosen individual [32]. While these metrics effectively address general diversity measurement goals, they do not directly align with our specific goal of evaluating the representation and coverage of minority subgroups. The $n^{th}$ nearest neighbor distance explicitly evaluates the distance between records in the unsampled group to those in the sampled group. This targeted focus enables a more precise assessment of the extent to which minority subgroups are included in study samples, thereby avoiding underrepresentation in the set of records used for CP development. As our real data analysis results showed, we can use the $n^{th}$ nearest neighbor distance to choose an optimal number of cluster k from which to sample. In particular, choosing too large of k leads to suboptimal performance.

Although the construction and generation of a chart review sample has not been widely discussed in the CP literature, parallel work exists in the active learning literature. Current active learning methods can be categorized as query-acquiring (pool-based) or query-synthesizing [20,33]. We focus on query-acquiring active learning, as query-synthesizing methods are not directly analogous to our work. Query-acquiring active learning uses various sampling strategies, including uncertainty sampling or information-theoretic measures, to identify which sampling strategies would be most impactful for continued labeling [18,34]. Therefore, the underlying burden for query-acquiring active learning is the same as in our method: an efficient need for labeling [35,36]. Most existing methods, including uncertainty sampling or information-theoretic measures, focus on identifying the most influential records to enhance the performance of a given prediction task [20,21,37-40]. In contrast, our method is not based on a supervised objective. Further, our method seeks to select records that best capture diversity, rather than records that are most representative. While representativeness and diversity may be related, they are not necessarily equivalent.

To illustrate our approach, we tested our method with the real-world task of identifying hospital encounters due to COVID-19. During the height of the COVID-19 pandemic (2020‐2023), all patients admitted to our health system’s hospitals were tested for SARS-CoV-2. As we and others have noted, approximately 38.2% of patients with a positive SARS-CoV-2 test were admitted for reasons other than COVID-19 [14,15]. Therefore, if one wanted to identify patients admitted due to COVID-19, a positive SARS-CoV-2 test would not be a sufficient CP because of its poor specificity. We compared the performance of the chart review sample based on a random selection of charts and our coverage sampling method. Overall, the coverage sample yielded a comparable performing CP. So, while coverage sampling did not yield better performance, the results conform to the simulation findings, which indicate that coverage is robust even when there is no underlying cluster structure or minority group.

While we selected charts based solely on clinical data elements, there were meaningful demographic differences between samples derived from randomly selected charts and through coverage sampling. For example, cluster 1 includes a much older patient population, with 50.2% (2959/5897) over the age of 65, compared to 25.3% (467/1846) in cluster 2. Patients in cluster 1 also have longer hospital stays (9.42 d on average) than those in cluster 2 (3.52 d). Additional details on cluster characteristics are provided in Table S1 in Multimedia Appendix 1. Consequently, the coverage sample exhibits a different distribution than the random sample. Specifically, the coverage sample included younger patients, a greater number of non-Hispanic Black patients (though fewer Hispanic patients), and more individuals with public insurance compared to the cohort derived from random sampling. This result highlights one of the key opportunities in this approach: deriving a less biased sample on which to build a CP. As others have described, one of the mechanisms of algorithmic bias is having unrepresentative samples used to develop the algorithm [41]. For instance, in the context of rare diseases, the typical ratio of patients with a given rare condition to those without the condition is approximately 100:1 [42,43]. In such scenarios, using random sampling may result in underrepresentation of minority subgroups in the chart review sample. When the review sample does not accurately reflect the patient population, the resulting CPs can produce biased results. For example, if certain demographic groups are underrepresented in the dataset, the CP may not learn to make accurate predictions for these groups, leading to disparities in performance [44]. To account for this, algorithmic solutions have been proposed, including data augmentation [45,46], resampling techniques [47,48], and algorithmic adjustments [49]. However, instead of addressing this problem algorithmically, we propose addressing it via design. As such, by clustering the data and sampling equally from the obtained clusters, we aim for the chart review sample to better represent the patient population. An advantage of our method is that it does not require the researcher to prespecify groups. Moreover, as our empirical results show, we are able to capture demographic diversity with just clinical data.

### Limitations

While our approach shows promise, there are some limitations. First, while our simulation results illustrate the potential improvement for coverage sampling, our real-world data example only showed nominal improvement. Further work should be conducted in other contexts. Second, the performance of our method is related to the quality of the cluster analysis. As others have noted, clustering methods can be highly variable [50]. This variability may be more obvious in EHR data, which often experience data quality issues. Because the clustering step is a means of obtaining a representative sample, we address this by generating multiple samples from multiple cluster structures and selecting the one with the best coverage. In principle, it is possible to skip the clustering step and directly choose an optimal sample, leading to more robust results. While such an approach is worthy of further exploration, it would be more computationally expensive and would not necessarily yield meaningfully better results. Third, while our study suggests that coverage sampling is not very sensitive to the choice of *n*, its robustness warrants further evaluation. Future researchers are encouraged to test a small range of *n* values (eg, n=1, 5, 10), as different choices of *n* may yield different samples. Another potential limitation is that the coverage sample (intentionally) generates a sample that will likely have a different event rate than the true event rate within the full patient population. While this does not present a problem for rank-based metrics like AUROC, it may affect the calibration of other metrics, such as Kullback-Leibler divergence [51]. When calibration is a priority, recalibration methods can be used [52].

### Conclusions

Overall, our results show that our coverage sampling method can provide a more representative sample than random sampling, especially when the source cohort contains minority subgroups. This approach can lead to the generation of a CP that has better performance in the overall study population as well as within subgroups. While CP development is a key part of secondary research with EHR data, little work has been done on how best to derive samples for learning CPs. This work addresses this gap and seeks to spur more investigation in this area. Ultimately, this sampling method has the potential to improve future clinical research by making gold-standard chart review labeling a more efficient process.

## Supplementary material

10.2196/72068 Multimedia Appendix 1 Supplemental materials regarding study results.

## Abbreviations

AUROC: area under the receiver operator characteristic curve
CP: computable phenotypes
EHR: electronic health record
