# Supplementary material for: Unsupervised Coverage Sampling to Enhance Clinical Chart Review Coverage for Computable Phenotype Development: Simulation and Empirical Study
Source: JMIR Med Inform. 2025 Nov 27;13:e72068. doi: 10.2196/72068 (PMC12661603; doi:10.2196/72068)
Supplement: Multimedia Appendix 1 [file medinform-v13-e72068-s001.docx]

**Supplementary Appendix**


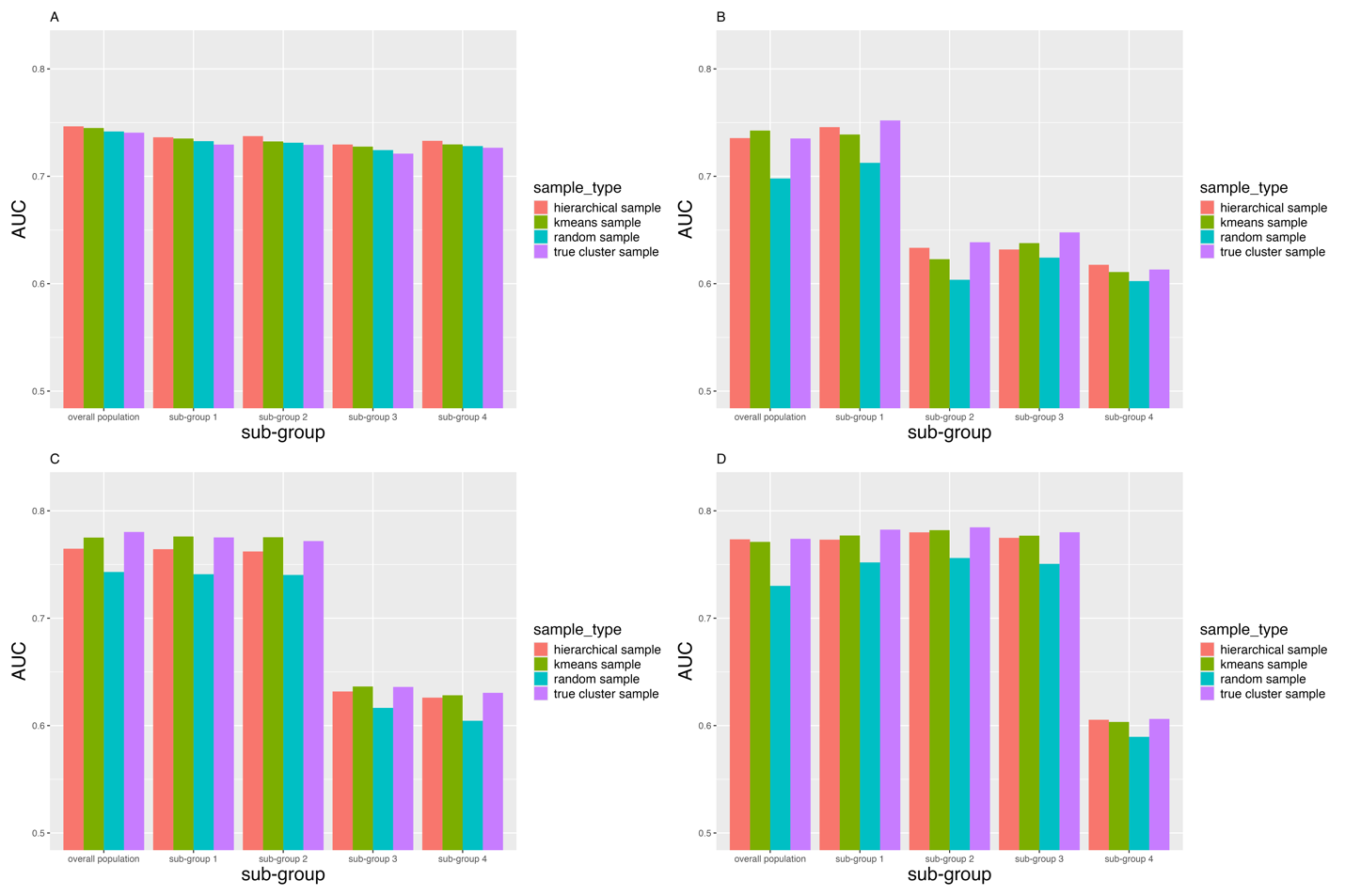


Figure S1: AUC comparison of no minority,1 minority,2 minority and 3 minority subcohort based on simulated data.

**Table S1: Demographic and clinical features’ Characteristics of COVID Dataset partitioned based on the 2-cluster structure.**

|  | Cluster 1 | Cluster 2 | SMD |
| --- | --- | --- | --- |
| Sample size | 5897 | 1846 |  |
| Testing_Time_Length_days (mean (SD)) | 1.82 (3.53) | 0.85 (0.90) | 0.378 |
| ADMIT_MONTH (mean (SD)) | 6.07 (3.87) | 5.79 (3.86) | 0.074 |
| ADMIT_YEAR (%) |  |  | 0.283 |
| 2020 | 1470 (24.9) | 291 ( 15.8) |  |
| 2021 | 1706 (28.9) | 485 ( 26.3) |  |
| 2022 | 2262 (38.4) | 936 ( 50.7) |  |
| 2023 | 459 ( 7.8) | 134 ( 7.3) |  |
| Length_Of_Stay_days (mean (SD)) | 9.42 (9.87) | 3.52 (3.47) | 0.798 |
| ENC_TYPE (%) |  |  | 0.702 |
| EI | 4798 (81.4) | 1362 ( 73.8) |  |
| IP | 662 (11.2) | 1 ( 0.1) |  |
| OS | 437 ( 7.4) | 483 ( 26.2) |  |
| group_discharge_disposition (%) |  |  | 0.921 |
| Dead | 714 (12.1) | 4 ( 0.2) |  |
| Home | 3922 (66.5) | 1815 ( 98.3) |  |
| Other Facility | 1201 (20.4) | 27 ( 1.5) |  |
| Others | 60 ( 1.0) | 0 ( 0.0) |  |
| ADMITTING_SOURCE (%) |  |  | 0.806 |
| AV | 392 ( 6.6) | 7 ( 0.4) |  |
| HO | 4354 (73.8) | 1833 ( 99.3) |  |
| IP | 696 (11.8) | 6 ( 0.3) |  |
| OT | 455 ( 7.7) | 0 ( 0.0) |  |
| group_primary_payment (%) |  |  | 0.183 |
| Others | 543 ( 9.2) | 114 ( 6.2) |  |
| Private | 2686 (45.5) | 878 ( 47.6) |  |
| Public | 2477 (42.0) | 738 ( 40.0) |  |
| Self-Pay | 191 ( 3.2) | 116 ( 6.3) |  |
| SEX = 1 (%) | 3004 (50.9) | 733 ( 39.7) | 0.227 |
| RACE_ETH (%) |  |  | 0.249 |
| HISPANIC | 531 ( 9.0) | 302 ( 16.4) |  |
| NHAsian | 74 ( 1.3) | 36 ( 2.0) |  |
| NHBlack | 2302 (39.0) | 697 ( 37.8) |  |
| NHwhite | 2827 (47.9) | 745 ( 40.4) |  |
| others | 163 ( 2.8) | 66 ( 3.6) |  |
| CRP_result (%) |  |  | 0.951 |
| High | 2908 (49.3) | 301 ( 16.3) |  |
| Normal | 437 ( 7.4) | 0 ( 0.0) |  |
| Not taken | 2552 (43.3) | 1545 ( 83.7) |  |
| D.Dimer_result (%) |  |  | 0.719 |
| High | 1395 (23.7) | 165 ( 8.9) |  |
| Normal | 2343 (39.7) | 388 ( 21.0) |  |
| Not taken | 2159 (36.6) | 1293 ( 70.0) |  |
| Ferritin_result (%) |  |  | 1.047 |
| High | 1889 (32.0) | 98 ( 5.3) |  |
| Low | 137 ( 2.3) | 0 ( 0.0) |  |
| Normal | 762 (12.9) | 19 ( 1.0) |  |
| Not taken | 3109 (52.7) | 1729 ( 93.7) |  |
| Procalcitonin_result (%) |  |  | 0.777 |
| High | 359 ( 6.1) | 0 ( 0.0) |  |
| Normal | 1160 (19.7) | 20 ( 1.1) |  |
| Not taken | 4378 (74.2) | 1826 ( 98.9) |  |
| ORIGINAL_BMI (%) |  |  | 0.539 |
| Normal | 1385 (23.5) | 349 ( 18.9) |  |
| Not Available | 689 (11.7) | 511 ( 27.7) |  |
| Obese | 2064 (35.0) | 597 ( 32.3) |  |
| Overweight | 1377 (23.4) | 388 ( 21.0) |  |
| Underweight | 382 ( 6.5) | 1 ( 0.1) |  |
| MEY_Notes_Anticoagulant.Antiplatelet = 1 (%) | 5335 (90.5) | 1143 ( 61.9) | 0.712 |
| MEY_Notes_Bronchodilator = 1 (%) | 1734 (29.4) | 207 ( 11.2) | 0.464 |
| MEY_Notes_Cough.Suppressant = 1 (%) | 1997 (33.9) | 323 ( 17.5) | 0.381 |
| MEY_Notes_Remdesivir = 1 (%) | 4369 (74.1) | 797 ( 43.2) | 0.661 |
| MEY_Notes_Inhaled.Steroid = 1 (%) | 836 (14.2) | 41 ( 2.2) | 0.447 |
| MEY_Notes_Paralytic = 1 (%) | 1900 (32.2) | 194 ( 10.5) | 0.549 |
| MEY_Notes_Steroid = 1 (%) | 2576 (43.7) | 369 ( 20.0) | 0.526 |
| MEY_Notes_Diuretic = 1 (%) | 1977 (33.5) | 259 ( 14.0) | 0.47 |
| MEY_Notes_Expectorant = 1 (%) | 1315 (22.3) | 177 ( 9.6) | 0.353 |
| MEY_Other = 1 (%) | 1235 (20.9) | 574 ( 31.1) | 0.233 |
| CCS_UP_52 = 1 (%) | 785 (13.3) | 41 ( 2.2) | 0.424 |
| CCS_UP_95 = 1 (%) | 197 ( 3.3) | 0 ( 0.0) | 0.263 |
| CCS_UP_98 = 1 (%) | 3247 (55.1) | 634 ( 34.3) | 0.426 |
| CCS_UP_130 = 1 (%) | 922 (15.6) | 64 ( 3.5) | 0.423 |
| CCS_UP_200 = 1 (%) | 110 ( 1.9) | 0 ( 0.0) | 0.195 |
| CCS_UP_60 = 1 (%) | 468 ( 7.9) | 10 ( 0.5) | 0.373 |
| CCS_UP_131 = 1 (%) | 605 (10.3) | 3 ( 0.2) | 0.467 |
| CCS_UP_163 = 1 (%) | 245 ( 4.2) | 1 ( 0.1) | 0.289 |
| CCS_UP_259 = 1 (%) | 1436 (24.4) | 113 ( 6.1) | 0.524 |
| CCS_UP_2620 = 1 (%) | 101 ( 1.7) | 0 ( 0.0) | 0.187 |
| CCS_UP_653 = 1 (%) | 586 ( 9.9) | 2 ( 0.1) | 0.462 |
| CCS_UP_245 = 1 (%) | 332 ( 5.6) | 0 ( 0.0) | 0.345 |
| CCS_UP_133 = 1 (%) | 1252 (21.2) | 190 ( 10.3) | 0.304 |
| CCS_UP_10 = 1 (%) | 241 ( 4.1) | 1 ( 0.1) | 0.286 |
| CCS_UP_195 = 1 (%) | 0 ( 0.0) | 168 ( 9.1) | 0.447 |
| CCS_UP_5 = 1 (%) | 92 ( 1.6) | 0 ( 0.0) | 0.178 |
| CCS_UP_84 = 1 (%) | 267 ( 4.5) | 6 ( 0.3) | 0.276 |
| CCS_UP_93 = 1 (%) | 247 ( 4.2) | 1 ( 0.1) | 0.29 |
| CCS_UP_127 = 1 (%) | 85 ( 1.4) | 0 ( 0.0) | 0.171 |
| CCS_UP_29 = 1 (%) | 92 ( 1.6) | 0 ( 0.0) | 0.178 |
| CCS_UP_79 = 1 (%) | 85 ( 1.4) | 0 ( 0.0) | 0.171 |
| CCS_UP_654 = 1 (%) | 84 ( 1.4) | 0 ( 0.0) | 0.17 |
| CCS_UP_657 = 1 (%) | 95 ( 1.6) | 0 ( 0.0) | 0.181 |
| CCS_UP_196 = 1 (%) | 0 ( 0.0) | 171 ( 9.3) | 0.452 |
| CCS_UP_Other = 1 (%) | 682 (11.6) | 156 ( 8.5) | 0.104 |
| AGE (%) |  |  | 0.725 |
| children | 211 ( 3.6) | 96 ( 5.2) |  |
| young adult | 425 ( 7.2) | 570 ( 30.9) |  |
| middle adult | 2302 (39.0) | 713 ( 38.6) |  |
| Old (>65) | 2959 (50.2) | 467 ( 25.3) |  |

*
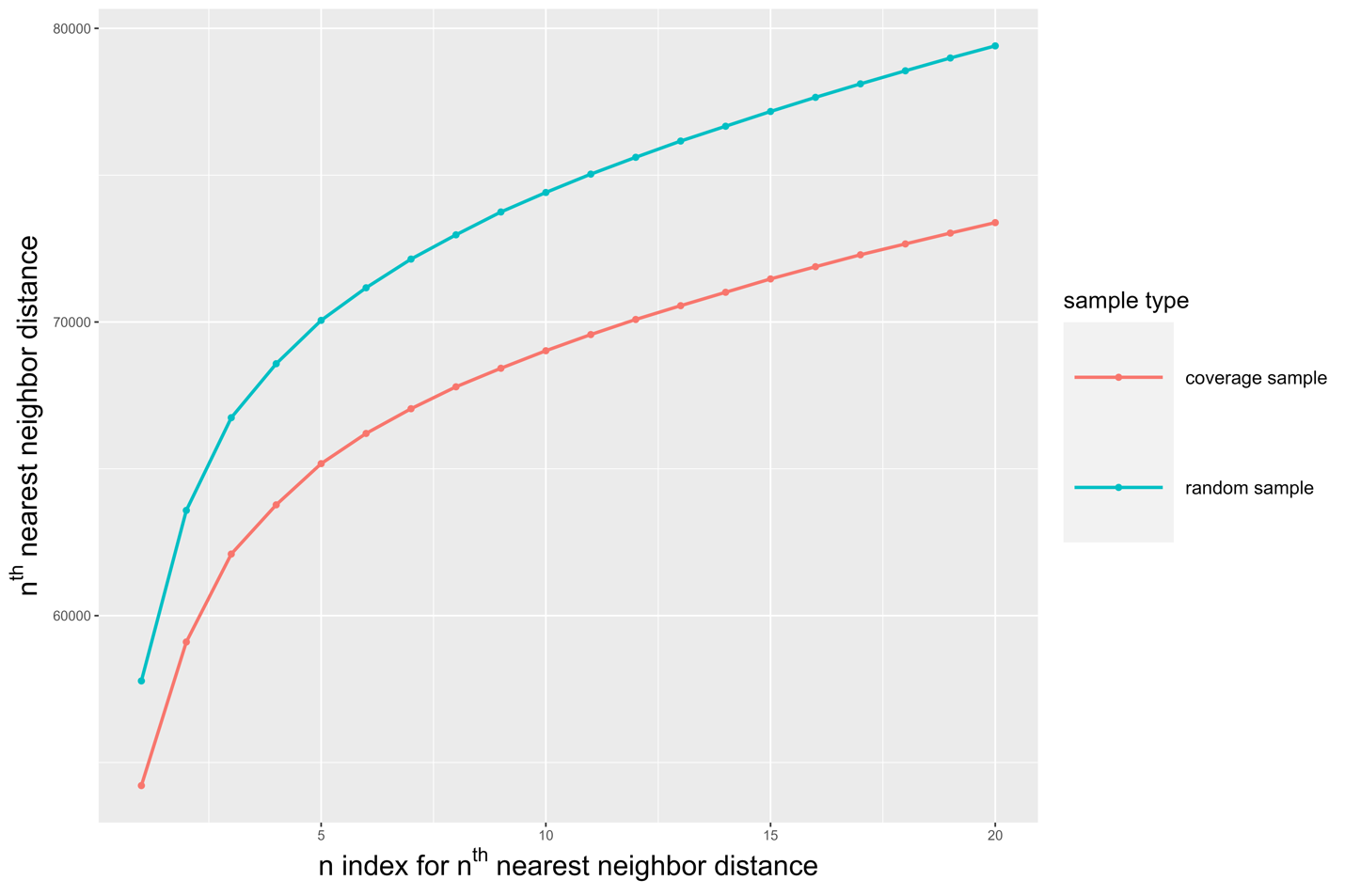
*

Figure S2: 1^st^ to 20^th^ nearest neighbor distance of real-world data random sample and coverage sample for real-world data.

**Table S2: AUROC comparison between random and coverage sampling across model architectures for real-world data**

| ***Model Arachitectures*** | ***Coverage Sample AUROC(95% CI)*** | ***Random Sample AUROC (95% CI)*** |
| --- | --- | --- |
| ***Random Forest*** | 0.788 (0.745,0.830) | 0.781 (0.738,0.825) |
| ***XGBoost*** | 0.759 (0.714,0.804) | 0.745 (0.699,0.791) |
